# On the Sr_1−*x*_Ba*_x_*FeO_2_F Oxyfluoride Perovskites: Structure and Magnetism from Neutron Diffraction and Mössbauer Spectroscopy

**DOI:** 10.3390/ma9120970

**Published:** 2016-11-29

**Authors:** Crisanto A. García-Ramos, María Retuerto, José Antonio Alonso

**Affiliations:** 1 Instituto de Ciencia de Materiales de Madrid, Consejo Superior de Investigaciones Científicas (C.S.I.C.), Cantoblanco, E-28049 Madrid, Spain; crisanto_garcia@yahoo.com (C.A.G.-R.); ja.alonso@icmm.csic.es (J.A.A.); 2 Institute for Nuclear Research and Nuclear Energy (I.N.R.N.E.), Tsarigradsko Chaussee 72, BG-1784 Sofía, Bulgaria; 3 Instituto de Catálisis y Petroleoquímica, Consejo Superior de Investigaciones Científicas (C.S.I.C.), Cantoblanco, E-28049 Madrid, Spain

**Keywords:** SrFeO_3−δ_, oxyfluoride, SrFeO_2_F, Sr_0.5_Ba_0.5_O_2_F, BaFeO_2_F, neutron diffraction, Mossbauer spectroscopy, antiferromagnetic structure

## Abstract

Four oxyfluorides of the title series (*x* = 0.00, 0.25, 0.50, 0.75) have been stabilized by topotactic treatment of perovskite precursors Sr_1−*x*_Ba*_x_*FeO_3−δ_ prepared by soft-chemistry procedures, yielding reactive materials that can easily incorporate a substantial amount of F atoms at moderate temperatures, thus avoiding the stabilization of competitive SrF_2_ and BaF_2_ parasitic phases. XRD and Neutron Powder Diffraction (NPD) measurements assess the phase purity and yield distinct features concerning the unit cell parameters’ variation, the Sr and Ba distribution, the stoichiometry of the anionic sublattice and the anisotropic displacement factors for O and F atoms. The four oxyfluorides are confirmed to be cubic in all of the compositional range, the unit cell parameters displaying Vergard’s law. All of the samples are magnetically ordered above room temperature; the magnetic structure is always G-type antiferromagnetic, as shown from NPD data. The ordered magnetic moments are substantially high, around 3.5 μ_B_, even at room temperature (RT). Temperature-dependent Mössbauer data allow identifying Fe^3+^ in all of the samples, thus confirming the Sr_1−*x*_Ba*_x_*FeO_2_F stoichiometry. The fit of the magnetic hyperfine field vs. temperature curve yields magnetic ordering *T*_N_ temperatures between 740 K (*x* = 0.00) and 683 K (*x* = 0.75). These temperatures are substantially higher than those reported before for some of the samples, assessing for stronger Fe-Fe superexchange interactions for these specimens prepared by fluorination of citrate precursors in mild conditions.

## 1. Introduction

The ideal ABX_3_ perovskite structure is usually described in a cubic unit cell (*a*_0_ ≈ 4 Å) with the B cations at the corners, the X anions at the midpoint of the edges and the larger A cations at the center of the cube. Whereas A is commonly any of a number of large ions (e.g., Ba, Sr, K, etc.), B is normally a relatively small transition metal or *p*-block element (e.g., Ti, Fe, Al, etc.), and X is usually oxygen; though many perovskites are known where X is fluoride, chloride, bromide or nitride [1]. Regarding the perovskite fluorides KMF_3_, most of them with M = Mg, V, Mn, Fe, Co, Ni, Zn, they all indeed exhibit a cubic structure [2]. For elements, such as Cu^2+^ with *d*^9^ configuration in the cubic crystal field, the perovskite KCuF_3_ is a Jahn–Teller (JT) active system, which lowers the crystal symmetry from the cubic structure. This compound has been treated as a prototype system in which the orbital ordering is induced by the superexchange interaction alone in the Kugel–Khomskii (KK) model [3]. The orbital ordering from this model results in a CuF_6_ octahedral-site distortion that consists of four long and two short Cu-F bonds.

Perovskites with a mixed anion sublattice, e.g., incorporating both O and F, are also well known and may present an attractive phenomenology. The fluorine incorporation in transition metal oxides, involving the alteration of the valence states of the transition metals, has been developed for a long time, mainly aiming to modify their electronic structures and, thus, change their electronic, transport and magnetic properties. In fact, the so-called oxyfluoride perovskites have attracted much attention since the discovery of superconductivity in copper perovskite-related oxyfluorides of the composition Sr_2_CuO_2_F_2__+_*_x_* [4].

The fluorination of the oxides has been mainly achieved by low-temperature techniques, due to the fact that the number of oxyfluorides that can be prepared by the standard high-temperature solid-state reaction is limited owing to the high stability of the simple fluorides used as starting materials. Therefore, low temperature reagents, such as F_2_ gas, NH_4_F, MF_2_ (M = Cu, Ni, Zn) or XeF_2_, have been utilized. Some sophisticated synthetic routes involve several steps, like the preparation of SrFeO_2_F starting with the SrFeO_3−__δ_ perovskite via the SrFeO_2_ infinite layer intermediate phase, finally reacting with XeF_2_ at 150 °C [5,6]. New, simpler synthesis procedures have also been developed that imply the heating of previously-formed oxide materials with a fluorinated polymer, poly(vinylidene fluoride). This method was successful for the synthesis of oxyfluorides as SrFeO_2_F, BaFeO_2_F, Ca_2_CuO_2_F_2_ or Sr_2_TiO_3_F_2_ [7,8,9,10]. The products obtained have high purity and crystallinity and do not show typical impurities as CaF_2_ or SrF_2_ that are usually present by using other fluorination techniques [11].

In this paper, we report on the synthesis of the series of compounds of general formula Sr_1−*x*_Ba*_x_*FeO_2_F (*x* = 0.00, 0.25, 0.50, 0.75). Some compounds of this series have been previously prepared by Clemens et al. [12]. By X-ray diffraction (XRD), they reported that these oxyfluorides are cubic perovskites in the whole range of *x*, with space group P*m-3m*. Furthermore, Mössbauer spectroscopic studies have been carried out for SrFeO_2_F, BaFeO_2_F and Sr_0.5_Ba_0.5_FeO_2_F [7,8,13,14]. In these works the materials were determined to contain a single Fe^3+^ oxidation state, and in the case of BaFeO_2_F, the valence of Fe was confirmed by Neutron Powder Diffraction (NPD) [8]. In the present work, we present complementary data; we determined the effects of the fluorination on the crystallographic and magnetic structures, studied by NPD, and on the magnetic properties of these appealing materials, in conjunction with a Mössbauer study.

## 2. Results

### 2.1. Crystallographic Characterization

**Figure 1:**
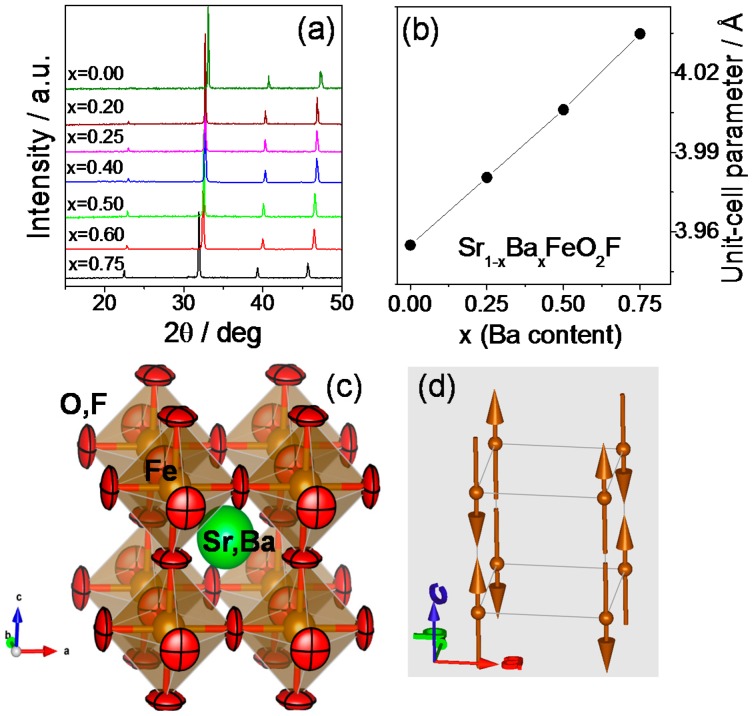
(**a**) X-ray diffraction (XRD) patterns (Cu Kα) for Sr_1−*x*_Ba*_x_*FeO_2_F (*x* = 0.00, 0.25, 0.50, 0.75). They all may be indexed in cubic P*m-3m* symmetry; (**b**) unit cell parameter variation with the Ba content (*x*); (**c**) view of the cubic crystal structure, displaying the anisotropic ellipsoids of (O,F) atoms, drawn with a 95% probability; (**d**) G-type antiferromagnetic structure determined from Neutron Powder Diffraction (NPD) data.

**Figure 2:**
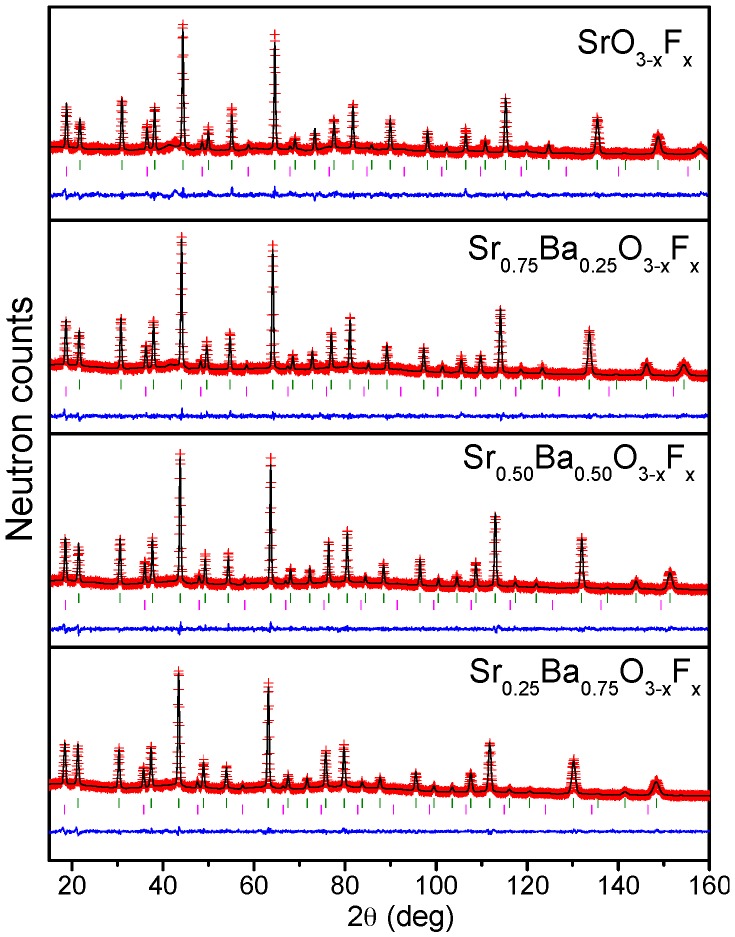
Observed (red crosses), calculated (black line) and difference of (bottom line) NPD Rietveld profiles for Sr_1−*x*_Ba*_x_*FeO_2_F (*x* = 0.00, 0.25, 0.50, 0.75) at room temperature (RT), obtained with λ = 1.594 Å. Green lines represent crystallographic Bragg reflections. Pink lines represent magnetic Bragg reflections.

Figure 1a shows the evolution of the XRD patterns. The diagrams show that the samples are crystalline and pure. As *x* increases, the reflections shift to lower angles, which mean larger unit cell parameters, as the content of Ba increases. To perform a careful structural study of the series and analyze the possible presence of vacancies, etc., we carried out a NPD study. Figure 2 illustrates the NPD data collected at room temperature, after the corresponding Rietveld refinement. All phases can be refined in P*m-3m* symmetry, as cubic perovskites. The cell parameters are in all cases greater than the compounds without fluorination. For instance, in the case of SrFeO_2_F, *a* = 3.95500(7) Å, larger than the unit cell parameter of stoichiometric SrFeO_3_ of 3.85086(4) Å [15], containing Fe^4+^ and prepared under rigorous high-pressure conditions. The same behavior is observed when Ba is introduced. This evolution is consistent with F incorporation, since the oxidation state of Fe concomitantly decreases, implying longer bond distances and cell parameters.

The evolution of the unit cell parameters along the series when Ba is introduced is shown in Figure 1b, displaying a linear increment with *x*, following Vergard’s law, since the driving force for the cell expansion is the difference in size of Ba^2+^ (1.61 Å in twelve-fold coordination) vs. Sr^2+^ (1.44 Å) [16]. The parameters obtained in all of the cases (Table 1) are comparable to those previously reported for fluorinated samples [12]. Unlike previous NPD determinations on SrFeO_2_F [6], our samples do not show the presence of spurious phases like SrF_2_, assessing our preparation procedure of (Sr,Ba)FeO_3−δ_ precursor perovskites, by citrate chemistry, as more suitable to obtain monophasic samples. It is noteworthy that the anisotropic refinement of the O/F displacement factors yields a substantial decrease of the *R*_Bragg_ discrepancy factor, e.g., from 5% down to 3% for *x* = 0.00. As shown in Figure 1c, the displacement ellipsoids for the oxygen atoms, drawn for 95% probability, are extremely flattened and can be described as disks perpendicular to the Fe-(O,F)-Fe bonding direction. This shape and configuration suggest strongly covalent Fe-(O,F) chemical bonds.

The oxygen/fluorine occupancy is virtually stoichiometric for the samples with larger Sr content; however, when the content of Ba increases, a small quantity of vacancies is detected. It is to be remarked that the introduction of F favors the anion stoichiometry of the phases: the precursor SrFeO_3−δ_ perovskite usually presents oxygen vacancies because Fe^4+^ is not favored, and it tends to partially reduce to Fe^3+^ when working in air or ambient pressure conditions; but the introduction of F implies the stabilization of Fe^3+^ without the need of anionic vacancies. The increment of the unit cell size upon Ba introduction implies a lengthening and concomitant weakening of the Fe-(O,F) bonds (Table 1), making possible the creation of a certain (small) proportion of anionic vacancies.

It is also worth mentioning that the actual symmetry of SrFeO_2_F has been the topic of some controversy, some authors indicating that it is cubic P*m-3m* [12], as mentioned before, and others report an orthorhombic I*mma* symmetry [18]. These authors show certain extra reflections in the diffraction patterns that arise from the reduction in symmetry to I*mma* (for instance [2 1 1]) that are not visible in our XRD or NPD data. Our diffraction data clearly assess for a cubic symmetry. In fact, their specimen reverts to cubic by warming up at moderate temperatures between 473 K and 523 K. It would seem that, depending on the preparation conditions, the symmetry of the compounds may vary, probably depending on subtle compositional variations, which are difficult to identify.

### 2.2. Magnetic Measurements

**Figure 3:**
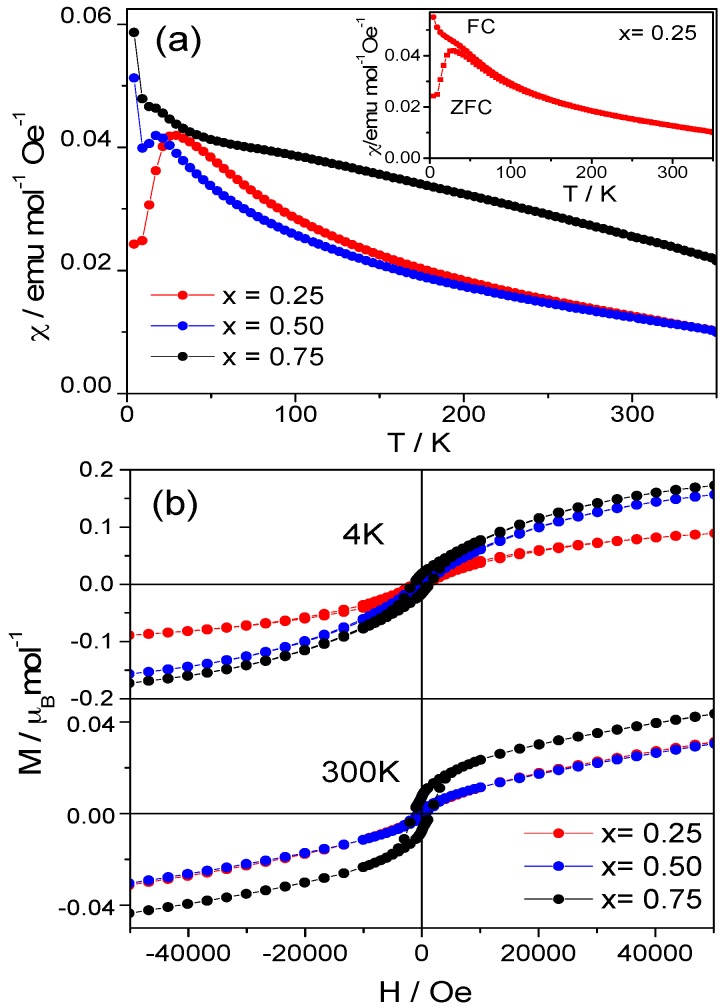
(**a**) Magnetic susceptibility vs. temperature for Sr_1−*x*_Ba*_x_*FeO_2_F; (**b**) magnetization vs. field. ZFC, Zero Field-Cooled.

The DC magnetic susceptibility vs. temperature has been measured for Sr_1−*x*_Ba*_x_*FeO_2_F (*x* = 0.25, 0.50 and 0.75) (Figure 3a). All of the compounds are magnetic at room temperature. ZFC (Zero Field-Cooled) and FC (Field-Cooled) DC magnetic susceptibility curves have been measured for all of the compounds, and a divergence between both curves is observed at low temperature, indicating magnetic frustration in the systems (inset, Figure 3a), which is well understood since in the presence of F, the geometric environment of the magnetic cations is no longer the same in all directions, so the interactions between spins is expected to be antagonistic. This divergence at low temperatures was previously described in former works, for instance for BaFeO_2_F [19], but the explanation in terms of a spin randomization was ruled out in subsequent studies [6] after the observation of a collinear G-type magnetic structure down to 4 K, proposing an alternative mechanism involving small volume spin-glassy domains or clusters.

Isothermal magnetization curves were measured at 4 K and 300 K. The curves are displayed in Figure 3b. The compounds have an antiferromagnetic behavior with some ferromagnetic interactions at both temperatures, which are concomitant with the low-temperature divergence described above. The net value of the magnetic moment decreases when the temperature increases. The antiferromagnetic response is still observed at RT, indicating that the magnetic ordering temperature is above RT.

### 2.3. Determination of the Magnetic Structure

The compounds are magnetic at RT, showing magnetic contributions on the NPD patterns collected at RT. The magnetic reflections appear on different positions than the crystallographic Bragg peaks. The magnetic structure can be explained using the propagation vector *k* = (½ ½ ½), indicating an antiferromagnetic system. There is only one magnetic cation in one position, Fe in (0,0,0), in both the crystallographic space group and P*-1* magnetic space group. Therefore, the magnetic structure can be defined as an antiferromagnetic configuration in the three space directions (G-type), with the moments oriented along the *c* axis (Figure 1d). The refinements of the magnetic structures at RT are shown as a second phase in Figure 2 (second line of Bragg reflections). The magnetic structure is the same for the four compounds under study. Table 1 shows the value of the Fe magnetic moments at RT. The values of the moments are very similar for all of the samples; all of them are around 3.5 μ_B_/mol, which is a high value considering that they have been measured at RT, and the expected magnitude for completely ordered Fe^3+^ ions (usually at low temperatures) is 5 μ_B_.

### 2.4. Mossbauer Analysis

In the analysis of the Mössbauer data, we have considered the situation where iron is surrounded by four O^2−^ ions and two F^−^ ions. The fluorine ion can be in two types of arrangements: *cis*-arrangement in which the two F ions are located at the adjacent corner; or *trans*, located at opposite corners of the octahedron. If the two F^−^ ions are in *cis*-arrangement, one of them is in the (0,½,0) position along the *y* axis, and the second in position (½,0,0) along the *x* axis, so the Electric Field Gradient (EFG) will have the direction of the bisector or diagonal <110>. Another alternative would be the equivalent cases considering that fluorine atoms are at (0,½,0) and (0,0,½) with EFG in diagonal <011>; or (0,−½,0) and (0,0,½) with EFG in diagonal <0-11>, etc. This diagonal will be the axis of maximum symmetry for the iron ion Fe^3+^. On the other side, for the *trans*-arrangement, we expect the axis of maximum symmetry to be along one of the coordinate axes, *x*, *y* or *z*, where F^−^-Fe^3+^-F^−^ are aligned along one of the crystallographic axis. For *cis*-/*trans*-arrangement, as we have a ratio of fluorine and oxygen 2F^−^:4O^2−^, it will give a 4:1 ratio of the peak area of Mossbauer spectra, since there are four more chances of having the *cis*- than the *trans*-disposition. The angle θ between the main EFG and magnetic hyperfine field *B*_hyp_ is given by the quadrupole splitting of the sextets by the expression: QS = ½ (eQV_zz_/4)(3sin^2^θ − 1) if the quadrupole splitting is treated as a perturbation of the hyperfine magnetic field, which causes the main splitting of the spectra. In the absence of magnetic field, when the temperature is greater than the magnetic order temperature, QS takes the value of ξ_0_ = eQV_zz_/4.

**Figure 4:**
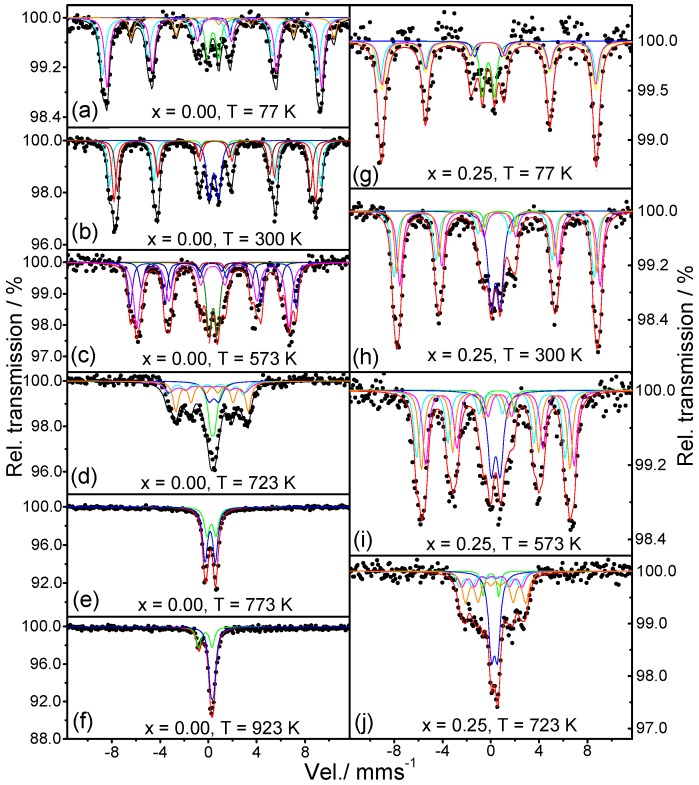
Mössbauer spectra for Sr_1−*x*_Ba*_x_*FeO_2_F: (**a**–**f**) *x* = 0.00 and (**g**–**j**) *x* = 0.25.

**Figure 5:**
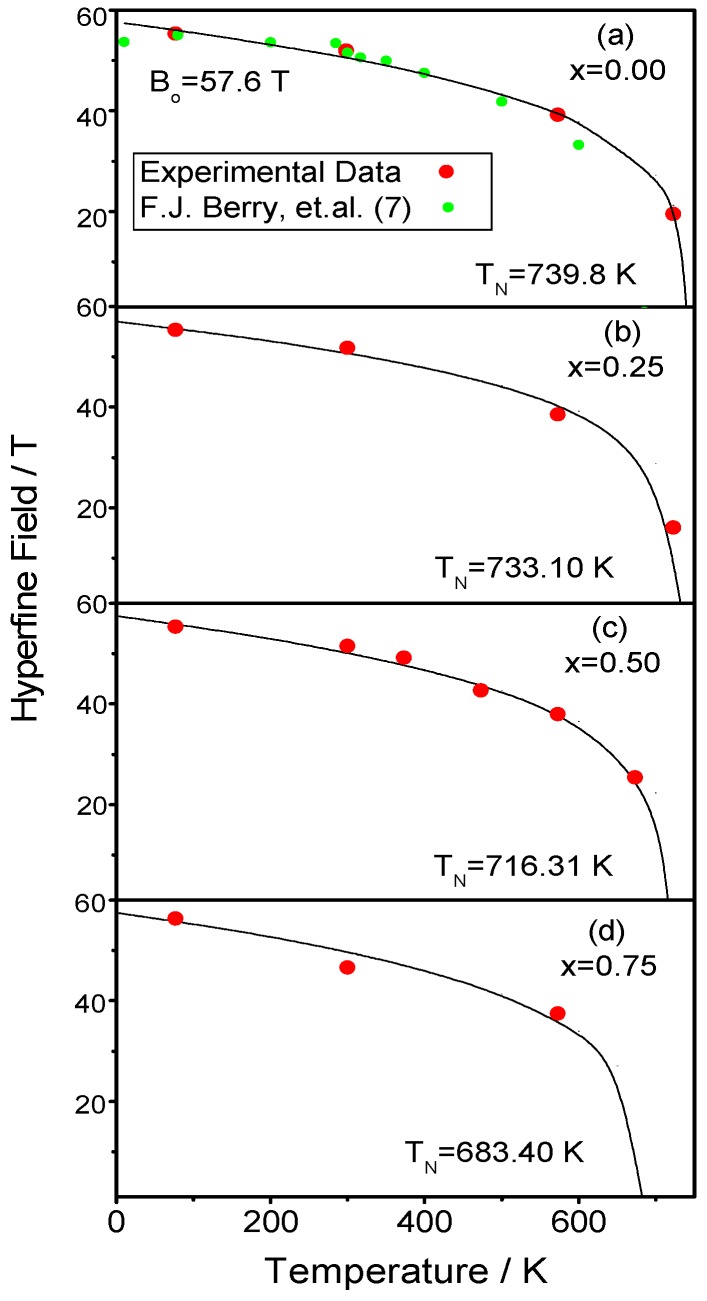
Dependence of the magnetic hyperfine field with the temperature for: (**a**) *x* = 0.00; (**b**) *x* = 0.25; (**c**) *x* = 0.50; and (**d**) *x* = 0.75.

The spectra of SrFeO_2_F (*x* = 0.00) between 77 K and 923 K are shown in Figure 4a–f, and the Mossbaüer parameters are listed in Table S1 (Supplementary Materials (SM)). The spectra from 77 K to 723 K clearly show two doublets and three sextets. The hyperfine magnetic field decreases with increasing the temperature from 77 K to 723 K. At 773 K, the spectrum shows only two doublets, indicating that the magnetic transition is located between 723 K and 773 K. From the doublets at 773 K, we obtain quadripolar Isomer Shifts (IS) characteristic of the presence of Fe^3+^ (Table S1). The variation of IS with the temperature is also consistent with that expected from the second order Doppler shift, which depends on the means square speed of lattice vibrations (*u*^2^), and indicates that IS decreases when the temperature increases. We have plotted the variation of the hyperfine magnetic field with the temperature (Figure 5a), and it is compared with the one calculated by the magnetic field expression:

*B* = *B*_o_(1 − *T*/*T*_N_)^α^ (1)

where *B*_o_ is the magnetic hyperfine field at 0 K (*B*_o_ = 57 T); α a parameter that is usually in the range 0.25 < α < 0.33; and *T*_N_ is the magnetic ordering temperature. In our calculation, we have taken α = 0.25 and the ^57^Fe Mossbauer data, yielding the parameters *B* = 55.5 T at 77 K; *B* = 1.9 T at 300 K; *B* = 39.1 T at 573 K; and *B* = 19.4 *T* at 723 K. Using these values in the previous formula, we obtain *T*_N_ = 739.8 K and *B*_o_ = 57.6 T. The value of *T*_N_ is higher than the previously reported *T*_N_ = 685 K [20] and the one determined by temperature-dependent NPD [6] located somewhere between 698 K and 723 K. This discrepancy can be related to a slightly different content of F (associated with the presence of SrF_2_ in the NPD patterns [6]) and a concomitantly higher oxidation state of Fe. In our measurements at 723 K (Figure 4d), we are still not in the paramagnetic region, in contrast with the spectrum previously reported at 700 K in [20], which already corresponds to a paramagnetic state. This is the case of our 773 K spectrum (Figure 4e) where two doublets are observed, corresponding to the paramagnetic phase. At 923 K (Figure 4f), there is only one doublet with a relative area of 29.90% and the singlet with a relative area of 70.08%, both characteristic of Fe^3+^.

**Figure 6:**
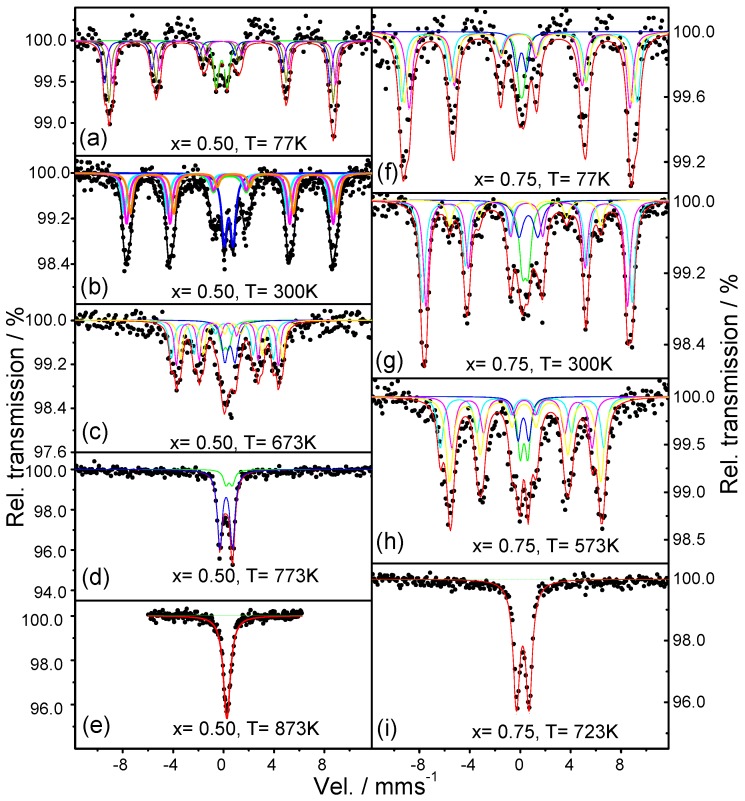
Mössbauer spectra for Sr_1−*x*_Ba*_x_*FeO_2_F: (**a**–**e**) *x* = 0.50 and (**f**–**i**) *x* = 0.75.

In the Sr_0.75_Ba_0.25_FeO_2_F (*x* = 0.25) spectra from 77 K to 723 K, we can clearly see a distribution of three magnetic sextets and two doublets (Figure 4g–j). The Mössbauer parameters are included in Table S2 (SM) and are characteristic of Fe^3+^. The changes of IS are consistent with the contribution resulting from the second order Doppler shift throughout the whole temperature range. The magnetic ordering temperature obtained from the evolution of the magnetic hyperfine field with temperature is 733.10 K (Figure 5b). The Mössbauer spectra recorded for *x* = 0.50 between 77 K and 873 K are shown in Figure 6a–e. The spectra show a distribution of three magnetic sextets and two doublets. The doublets are again characteristic for Fe^3+^ (Figure 6b; Table S3). The spectrum at 773 K (Figure 6d) shows two doublets indicating a paramagnetic state. The spectrum at 873 K corresponds to a singlet. Using the data obtained in Table S3, we found that the hyperfine magnetic field at 0 K is *B*_hyp_ = 57.85 T, and the Neel temperature *T*_N_ = 716.31 K (Figure 5c), so that at 673 K, we still see a magnetic compound, again finding a discrepancy with previously-published results, where magnetic order temperature is *T*_N_ ~ 670 (±10) K [21]. In the case of *x* = 0.75, the evolution of the spectra is very similar to previous samples. The change from magnetic to paramagnetic state is observed between 573 K and 723 K (Figure 6f–i). The obtained IS and hyperfine fields (Table S4) are also characteristic of Fe^3+^, and the changes of IS are consistent with the contribution resulting from the second order Doppler shift. The variations of the average magnetic hyperfine field with temperature gives the hyperfine magnetic field at 0 K, *B*_hyp_ = 57.50 T and the Néel temperature of *T*_N_ = 683.4 K, as shown in Figure 5d.

## 3. Discussion

We have prepared four oxyfluorides of the series Sr_1−*x*_Ba*_x_*FeO_2_F (*x* = 0.00, 0.25, 0.50, 0.75). Good quality NPD data show that Sr and Ba occupy at random the a positions of the perovskite, and O and F are randomly distributed over the anionic sublattice, with no oxygen/fluorine vacancies (*x* = 0.00, 0.25) or moderate values for large Ba occupancies (*x* = 0.50, 0.75), where Fe-(O,F) bonds are weakened. For the cations (Sr,Ba,Fe), the thermal displacement parameters are constrained by symmetry to be spherical. For O, the anisotropy of the thermal ellipsoids is patent, with the smallest thermal motions along the Fe-(O,F) bonds. The O oblate ellipsoids, flattened along the Fe-(O,F)-Fe directions, are oriented along the [001] directions (Figure 1c). For instance, for SrFeO_2_F, the root mean square (r.m.s.) displacements of O are 0.206 Å perpendicular to the Fe-Fe direction and 0.089 Å parallel to it. The disk-shaped ellipsoids are the result of the strong covalent bonding between Fe^3+^ and O^2−^ or F^−^ by virtue of the robust covalent mixing between 3d Fe orbitals and (O,F) 2p orbitals, strongly overlapping across 180° Fe-(O,F)-Fe angles. Such strong chemical bonds impede the thermal motion along the bonds, in such a way that (O,F) atoms exhibit degrees of freedom in the plane perpendicular to the bonding direction. 

Although only NPD collected at RT are available, the robustness of the magnetic structure made possible a full refinement of the Fe magnetic moments, confirming the occurrence of a G-type antiferromagnetic collinear structure for the four different compositions described here, attaining similarly-ordered magnetic moments for Fe between 3.4 μ_B_ and 3.6 μ_B_, considerably high at room temperature. No symptoms of non-collinear ordering were observed, which could have accounted for the divergence between ZFC and FC susceptibility curves at low temperatures or the bending of the magnetization isotherms at 4 K and even at 300 K. It must be mentioned that the saturation magnetization at 300 K is not reached even at external magnetic fields of 5 T, but the extrapolation to *H* = 0 would give a weak remnant field of ~0.02 μ_B_, not observable by NPD as an extra contribution on the Bragg peaks.

The analysis of the Mössbauer spectra for the four compositions consistently indicates the presence of Fe^3+^ ions in all of the samples, confirming the Sr_1−*x*_Ba*_x_*FeO_2_F stoichiometry. In the magnetically-ordered region, the observation of three sextets is to be expected from the random distribution of 4 O and 2 F anions averaged over the perovskite octahedra, collapsing into two doublets when the Néel temperature is overcome, above room temperature. The magnetic ordering temperature decreases from 739.8 K (*x* = 0.00) to 715.70 K (*x* = 0.50) and 683.4 K (*x* = 0.75), as a result of the lengthening of the Fe-(O,F)-Fe superexchange paths. One important result distilled from the Mössbauer data is that the determined *T*_N_’s are substantially higher that those reported for specimens of comparable compositions SrFeO_2_F (710 K, [6]), Sr_0.5_Ba_0.5_FeO_2_F (670 K, [14]), as commented above. These results indicate better superexchange interactions in the present samples that could arise from a stricter Sr_1−*x*_Ba*_x_*FeO_2_F stoichiometry and Fe^3+^ contents, originated by the original synthesis procedure starting from citrate precursors for the formation of the perovskite oxide networks. It must be recalled that literature samples often contain significant SrF_2_ and BaF_2_ parasitic, competitive phases.

**Figure 7:**
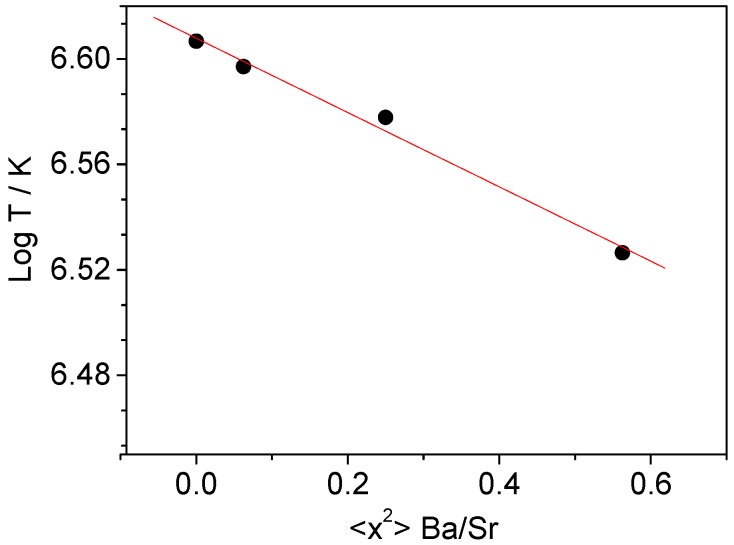
Variation of the Néel temperature with *x* across the Sr_1−*x*_Ba*_x_*FeO_2_F series.

A further analysis of the *T*_N_ variation with the Ba contents (*x*) shows that the temperature has an exponential dependence on *x*^2^ (Figure 7). We obtained the expression LnT = *A* + *Bx*^2^, where *A* and *B* are constants determined as *A* = 6.607(2) and *B* = −0.138(4). To explain this dependence, it is necessary to take into account the commented on dependence of the lattice parameter with *x* (Figure 1b). The lattice parameter is given by the expression *a* = ∑*x*_k_(*V*_k_/*Z*_k_)^δ^ with *x*_k_ = refined mole fraction of phase k, *V*_k_ = unit cell volume of k, *Z*_k_ = number of formula unit per unit cell for phase k and δ = 1/3; *N* = the number of phases. As the unit cell parameter *a* depends on *x*, the Néel temperature will also depend on *a*. To find the relation between the two variables, we need to express the dependence of *x* with *a* as *k* = (*a* − *a***_0_**)/*x*; the slope of the curve a versus *x* determines *k*, with *a***_0_** = 3.95500(7) Å, *k* = 0.105924. The dependence of the temperature of lattice parameter a may be expressed as LnT_N_ = *A* + *E*(*a* − *a***_0_**)^2^, with *E* = −12.277. For our perovskite-type Sr_1−*x*_Ba*_x_*FeO_2_F compounds, the simplified expression for the magnetic order temperature depending of lattice parameter *a*, expressed in angstroms, will be: *T*(*a*) = e^6.6079^ × exp{−12.277(*a* − 3.95500)^2^}. If we take into account the main bond distance Fe-(O,F), 2*d*_0_ = *a*_0_, the expression for temperature vs. main bond distance will be: *T*_N_ = e^6.6079^ × exp{−49.108(*d* − 1.97750)^2^}. Using the relation $\mathrm{LnT}=A+Bx^{2}$ we may predict the magnetic order temperature for a whole series of Sr_(1−*x*)_Ba_(*x*)_FeO_2_F. For instance, for *x* = 1.00 (BaFeO_2_F) *T*_N_ is predicted as 644.99 K, which is very close to the experimental value of 645.5 K. [19].

## 4. Materials and Methods

### 4.1. Synthesis

Sr_1−*x*_Ba*_x_*FeO_3−δ_ (*x* = 0.00, 0.25, 0.50, 0.75) precursor oxides were prepared by a wet chemistry procedure. This method requires the formation of very reactive precursors starting from an aqueous solution of the metal ions and citric acid. Stoichiometric amounts of Sr(NO_3_)_2_, BaCO_3_ and FeC_2_O_4_·2H_2_O were dissolved in citric acid and some drops of nitric acid, and the solution was slowly dehydrated by gentle heating, leading to organic resins that contain a homogeneous distribution of the involved cations. The formed resins were dried at 120 °C, decomposed at 600 °C for 12 h, and the organic materials and the nitrates were eliminated in a subsequent treatment at 800 °C in air, for 2 h. This treatment gave rise to finely-divided and homogeneous precursor materials that finally were heated in air at 1000 °C for 12 h to obtain the pure perovskite oxide precursor phases. 

The fluorination of the samples was carried out by a low-temperature topotactic reaction, keeping unchanged the backbone of the perovskite structure and replacing a limited number of O by F anions. The pristine oxides were mixed and thoroughly ground with poly(vinylidene fluoride) in a 1:0.75 molar ratio, and the mixture was heated at 380 °C for 24 h in flowing nitrogen.

### 4.2. X-ray Diffraction and Neutron Diffraction Data

The initial characterization was performed by XRD using a Bruker-axs D8 Advanced diffractometer (40 kV, 30 mA), controlled by DIFFRACT^PLUS^ software in Bragg–Brentano reflection geometry with Cu Kα radiation (λ = 1.5418 Å) and a Position Sensitive Detector (PSD). A filter of nickel allows the complete removal of Cu Kβ radiation. The slit system was selected to ensure that the X-ray beam was completely within the sample for all 2θ angles. The data were obtained between 10° and 70° 2θ in steps of 0.05°.

NPD experiments of the fluorinated samples were carried out in a High-Resolution Powder diffractometer for Thermal neutrons (HRPT) (λ = 1.494 Å) at room temperature, at the Laboratory for Neutron Scattering, Paul Scherrer Institute, Switzerland. NPD data were analyzed by the Rietveld method using the Fullprof program [17,21]. A pseudo-Voigt function was chosen to generate the line shape of the diffraction peaks. The following parameters were refined in the final run: scale factor, background coefficients, zero-point error, pseudo-Voigt corrected for asymmetry parameters, positional coordinates and isotropic thermal factors for all of the atoms. 

### 4.3. DC Magnetic Susceptibilities

The magnetic measurements were performed in a commercial superconducting quantum interference device magnetometer. ZFC and FC DC magnetic susceptibility data were collected in the 5 K ≤ *T* ≤ 400 K range under an applied magnetic field of 1000 Oe. Isothermal magnetization curves were obtained for magnetic fields going from −5 T to 5 T at *T* = 5 K and 300 K.

### 4.4. Mössbauer Spectroscopy

The Mössbauer spectra have been recorded in transmission mode using a conventional spectrometer with a ^57^Co/Rh source. To avoid saturation effects and to optimize the signal to noise ratio, the sample thickness was 10 mg of natural Fe/cm^2^. The analyses of the spectra were made by a nonlinear fit using the MIF-Mossbauer integral fit program-based on the properties of an integral Lorentzian line shape approximation [22,23,24]. This approach gives the possibility to separate the absorber line widths (scattered) and avoid the saturation thickness effect, and the energy calibration was made with α-Fe (6 μm) foil. A NB sample holder was used for high temperature measurements, while another one with Be windows was used in the low temperature range. The ^57^Fe Mossbauer spectra of the Sr_1−*x*_Ba*_x_*FeO_2_F (*x* = 0.00, 0.25, 0.50, 0.75) compounds was recorded at 77 K using liquid nitrogen. The spectra were also recorded at room temperature (300 K, RT), 373 K, 473 K, 573 K, 673 K, 723 K, 773 K, 823 K, 873 K and and 923 K, using an appropriate specially-designed furnace. The ^57^Fe Mossbauer chemical IS data were quoted to iron at RT [22].

## 5. Conclusions

Four metastable phases, obtained by topotactic fluorination of perovskite oxide networks at moderate temperatures, have been evaluated by neutron diffraction, showing distinct features, relating to the phase symmetry, purity, anisotropic displacement factors and anionic stoichiometry. The magnetic structure is G-type antiferromagnetic for the four oxyfluorides, consistent with that described previously for SrFeO_2_F. The divergence observed between FC and ZFC susceptibility curves and the ferro-(ferri-)magnetic component observed in the magnetization isotherms suggest a subtle canting of the magnetic moments (even at room temperature) that cannot be observed from NPD data. The Mössbauer spectra for the four compounds are consistent with the presence of Fe^3+^; as expected, the magnetic order temperature decreases as the ratio of Sr/Ba increases, since the superexchange interactions between Fe^3+^ neighbors are weakened as the unit cell size increases upon Ba^2+^ incorporation into the lattice. This dependence may be expressed as: *T*(*x*) = exp{6.6079 − 0.13775*x*^2^}, where *x* is the Ba content. It is remarkable that the observed *T*_N_’s are significantly higher than those reported before; we suggest that this is a result of the better quality and optimized Fe^3+^ content of our samples.

## Figures and Tables

**Table 1:** Structural parameters for Sr_1−*x*_Ba*_x_*FeO_3−*y*_F*_y_*, refined in the cubic P*m-3m* space group, from Neutron Powder Diffraction (NPD) data at room temperature (RT). Reliability factors are also given.

***x***	0	0.25	0.50	0.75
***a* (Å)**	3.95500(7)	3.98055(6)	4.00610(5)	4.03476(6)
***V* (Å^3^)**	61.864(2)	63.071(2)	64.293(1)	65.683(2)
**Sr/Ba 1b (½ ½ ½)**				
**B (Å^2^)**	0.80(4)	0.81(3)	0.87(3)	0.84(3)
**Fe 1a (0 0 0)**				
**B (Å^2^)**	1.62(4)	1.89(3)	2.29(3)	2.68(3)
**μ_B_**	3.63(4)	3.50(3)	3.37(3)	3.40(2)
**O/F 3d (0 0 ½)**				
**B (Å^2^)**	2.36(4) *	1.55(3)	1.30(3)	1.10(2)
**Occupancy O/F**	1	1	0.98(1)	0.96(1)
**Main bond distances (Å)**				
**Sr-O/F (×12)**	2.79661(4)	2.81467(3)	2.83274(2)	2.85301(3)
**Fe-O/F (×6)**	1.97750(4)	1.99028(3)	2.00305(2)	2.01738(3)
Reliability factors **				
**χ^2^**	1.78	1.39	1.33	1.35
***R*_p_ (%)**	3.46	2.71	2.87	2.12
***R*_wp_ (%)**	4.34	3.41	3.63	2.69
***R*_exp_ (%)**	3.25	2.89	3.15	2.31
***R*_I_ (%)**	4.91	2.60	2.01	1.53
***R*_mag_ (%)**	17.14	8.51	7.12	6.09

* Anisotropic Betas (×10^4^). β_11_ = β_22_ = 535(11), β_33_ = 99(13); β_12_ = β_13_ = β_23_ = 0. ** The reliability factors χ^2^, *R*_p_, *R*_wp_, *R*_exp_, *R*_I_ and *R*_mag_ are defined in Reference [17].

## Supplementary Materials

The following are available online at www.mdpi.com/1996-1944/9/12/970/s1. Table S1: Mössbauer data for SrFeO_2_F; Table S2: Mössbauer data for Sr_0.75_Ba_0.25_FeO_2_F; Table S3: Mössbauer data for Sr_0.5_Ba_0.5_FeO_2_F; Table S4: Mössbauer data for Sr_0.25_Ba_0.75_FeO_2_F.
