# Supplementary material for: On the Sr1−xBaxFeO2F Oxyfluoride Perovskites: Structure and Magnetism from Neutron Diffraction and Mössbauer Spectroscopy
Source: Materials (Basel). 2016 Nov 29;9(12):970. doi: 10.3390/ma9120970 (PMC5456977; doi:10.3390/ma9120970)
Supplement: Supplementary file 1 [file materials-09-00970-s001.pdf]

Supplementary Materials: On the $Sr_{1-x}Ba_xFeO_2F$ Oxyfluoride Perovskites: Structure and Magnetism from Neutron Diffraction and Mössbauer Spectroscopy

Crisanto A. García-Ramos, María Retuerto and José Antonio Alonso

Mössbauer Spectroscopy Tables

Table S1. Mössbauer data for $SrFeO_2F$. *Q* Split: Quadrupolar splitting. B_{hyp}: hyperfine magnetic field. FWHM: Full width at half maximum.

Temperature		Isom Shift (mm·s⁻¹)	*Q* Split (mm·s⁻¹)	FWHM (mm·s⁻¹)	B_{hyp} (T)	Area Peak (%)
77 K	Sextet	0.300	+0.050	0.470	56.19	37.31
	Sextet	0.390	+0.050	0.400	54.11	38.81
	Sextet	2.100	0.000	0.370	52.24	11.94
	Doublet	0.382	0.946	0.370	-	10.41
	Doublet	−0.100	1.500	0.200	-	1.49
300 K	Sextet	0.500	−0.080	0.570	53.64	26.85
	Sextet	0.480	0.080	0.300	51.62	27.74
	Sextet	0.500	0.100	0.470	50.06	26.85
	Doublet	0.500	1.800	0.400	-	0.67
	Doublet	0.470	0.780	0.450	-	17.90
573 K	Sextet	0.300	−0.080	0.500	41.64	20.73
	Sextet	0.300	0.070	0.470	39.18	38.86
	Sextet	0.300	0.060	0.470	35.88	18.13
	Doublet	0.250	1.300	0.290	-	3.11
	Doublet	0.350	0.600	0.590	-	19.17
723 K	Sextet	0.000	−0.073	0.600	18.65	17.24
	Sextet	0.300	0.000	0.550	18.65	31.03
	Sextet	0.100	−0.011	0.970	16.32	20.69
	Doublet	0.380	0.200	0.470	-	17.24
	Doublet	0.447	0.800	0.400	-	13.79
773 K	Doublet	0.250	0.750	0.45	-	35.48
	Doublet	0.150	0.850	0.40	-	64.52
923 K	Doublet	−0.250	1.100	0.400	-	29.92
	Doublet	0.320	-	0.380	-	70.08

Table S2. Mössbauer data for $Sr_{0.75}Ba_{0.25}FeO_2F$.

Temperature		Isom Shift (mm·s⁻¹)	*Q* Split (mm·s⁻¹)	FWHT (mm·s⁻¹)	B_{hyp} (T)	Area Peak (%)
77 K	Sextet	−0.241	+0.050	0.47	56.07	24.92
	Sextet	−0.210	+0.050	0.47	55.04	24.92
	Sextet	−0.210	+0.050	0.57	55.04	32.71
	Doublet	−0.200	1.000	0.40	-	13.55
	Doublet	−0.200	2.250	0.40	-	3.89
300 K	Sextet	0.490	0.000	0.47	53.27	27.03
	Sextet	0.650	0.000	0.47	50.37	30.41
	Sextet	0.500	0.000	0.37	51.62	20.27
	Doublet	0.800	2.600	0.27	-	2.03
	Doublet	0.400	0.750	0.57	-	20.27

Table S2. *Cont.*

Temperature		Isom Shift (mm·s^{-1})	*Q* Split (mm·s^{-1})	FWHT (mm·s^{-1})	B_{hyp} (T)	Area Peak (%)
573 K	Sextet	0.200	0.000	0.47	40.83	24.59
	Sextet	0.240	0.000	0.50	35.73	28.69
	Sextet	0.400	−0.050	0.47	38.87	27.87
	Doublet	0.650	2.600	0.27	-	2.46
	Doublet	0.400	0.750	0.60	-	16.39
723 K	Sextet	0.036	0.000	0.57	17.13	15.63
	Sextet	0.100	0.000	0.57	15.54	15.63
	Sextet	0.073	0.000	0.57	15.54	31.25
	Doublet	0.000	1.300	0.27	-	6.25
	Doublet	0.300	0.500	0.50	-	31.25

Table S3. Mössbauer data for $Sr_{0.5}Ba_{0.5}FeO_2F$.

Temperature		Isom Shift (mm·s^{-1})	*Q* Split (mm·s^{-1})	FWHT (mm·s^{-1})	B_{hyp} (T)	Area Peak (%)
77 K	Sextet	−0.224	0.000	0.370	57.22	23.94
	Sextet	−0.200	0.000	0.370	55.35	35.21
	Sextet	−0.128	0.000	0.370	53.48	23.94
	Doublet	−0.160	0.865	0.370	-	8.45
	Doublet	−0.150	0.730	0.370	-	8.45
300 K	Sextet	0.520	−0.100	0.370	53.48	21.05
	Sextet	0.400	0.000	0.370	49.44	21.05
	Sextet	0.520	−0.500	0.350	51.62	32.89
	Doublet	0.400	1.300	0.370	-	9.21
	Doublet	0.500	0.800	0.370	-	15.79
373 K	Sextet	0.558	0.0175	0.400	51.06	26.56
	Sextet	0.500	0.000	0.400	48.98	26.56
	Sextet	0.540	0.095	0.400	47.45	28.12
	Doublet	0.150	0.700	0.370	-	3.13
	Doublet	0.450	0.750	0.370	-	15.63
473 K	Sextet	0.490	0.100	0.370	46.02	27.40
	Sextet	0.500	0.080	0.300	44.47	23.29
	Sextet	0.420	0.150	0.370	42.72	27.40
	Doublet	0.200	0.600	0.470	-	8.22
	Doublet	0.550	0.900	0.400	-	13.70
573 K	Sextet	0.320	−0.050	0.470	39.33	16.81
	Sextet	0.350	−0.050	0.470	37.94	26.55
	Sextet	0.300	−0.050	0.470	36.54	23.01
	Doublet	0.150	0.500	0.470	-	9.02
	Doublet	0.500	0.800	0.470	-	10.62
673 K	Sextet	0.240	−0.050	0.470	27.64	24.00
	Sextet	0.300	0.050	0.400	25.19	25.60
	Sextet	0.240	0.050	0.400	23.04	25.60
	Doublet	0.150	0.450	0.470	-	9.60
	Doublet	0.500	0.750	0.570	-	15.20
773 K	Doublet	0.190	0.850	0.450	-	50.00
	Doublet	0.150	1.000	0.450	-	50.00
823 K	Doublet	0.210	0.200	0.470	-	50.00
	Doublet	0.110	0.800	0.420	-	50.00
873 K	Singlet	0.260	-	0.570	-	100.00

Table S4. Mössbauer data for $Sr_{0.25}Ba_{0.75}FeO_2F$.

Temperature		Isom Shift (mm·s^{-1})	*Q* Split (mm·s^{-1})	FWHT (mm·s^{-1})	B_{hyp} (T)	Area Peak (%)
77 K	Sextet	−0.100	0.050	0.470	58.15	26.51
	Sextet	−0.100	0.050	0.470	54.51	28.92
	Sextet	−0.100	−0.050	0.570	56.28	31.33
	Doublet	+0.100	0.300	0.400	-	7.23
	Doublet	+0.100	0.800	0.400	-	6.02
300 K	Sextet	0.540.	0.050	0.420	51.74	34.86
	Sextet	0.470	0.000	0.400	49.44	33.33
	Sextet	0.300	0.095	0.450	37.31	9.17
	Doublet	0.400	0.450	0.500	-	11.93
	Doublet	0.650	1.500	0.700	-	9.17
573 K	Sextet	0.230	−0.086	0.470	40.36	21.16
	Sextet	0.230	−0.086	0.470	34.58	21.16
	Sextet	0.350	0.040	0.570.	37.31	40.21
	Doublet	0.400	0.600	0.400	-	10.05
	Doublet	0.650	0.850	0.400	-	7.41
723 K	Doublet	0.200	1.000	0.500	-	100.00
